# Antitumor Effects of PRMT5 Inhibition in Sarcomas

**DOI:** 10.1158/2767-9764.CRC-23-0239

**Published:** 2023-11-02

**Authors:** Stéphanie Verbeke, Aurélien Bourdon, Jean-Philippe Guegan, Laura Leroy, Vanessa Chaire, Elodie Richard, Alban Bessede, Antoine Italiano

**Affiliations:** 1 Sarcoma Unit, Bergonié Institute, Bordeaux, France.; 2 INSERM U1312 BRIC BoRdeaux Institute of onCology, University of Bordeaux, Bordeaux, France.; 3 Explicyte, Bordeaux, France.; 4 Service Commun des Animaleries, University of Bordeaux, Bordeaux, France.; 5 Faculty of Medicine, University of Bordeaux, Bordeaux, France.

## Abstract

Patients with advanced soft-tissue sarcomas (STS) have few therapeutic options. Protein arginine methyltransferase 5 (PRMT5), an anticancer target, has been extensively investigated in recent years in epithelial tumors. To date, no data related to the biological role of PRMT5 inhibition and its potential effect as a treatment in STS have been reported.

To investigate the therapeutic potential of PRMT5 targeting in STS, we first evaluated the prognostic value of *PRMT5* expression in two different cohorts of patients with STS. We then used the potent and selective GSK3326595 (GSK595) compound to investigate the antitumor effect of the pharmacologic inhibition of PRMT5 *in vitro* via MTT, apoptosis, cell cycle, clonogenicity, and proliferation assays. *In vivo* studies were performed with two animal models to evaluate the effects of GSK595 on tumor growth. The mechanisms of action were investigated by RNA sequencing, metabolic pathway analysis, Western blotting, and glucose uptake/lactate production assays.

High *PRMT5* gene expression levels were significantly associated with worsened metastasis-free survival of patients with STS. GSK595 decreased the global symmetric dimethylarginine level, the proliferation rate and clonogenicity of STS cell lines *in vitro* and tumor growth *in vivo*. Moreover, PRMT5 inhibition regulated aerobic glycolysis through downregulation of key enzymes of glycolysis as well as glucose uptake and lactate production.

The current study demonstrated that PRMT5 regulates STS cell metabolism and thus represents a potential therapeutic target for STS. Additional studies in diverse sarcoma subtypes will be essential to confirm and expand upon these findings.

**Significance:**

STSs have limited therapeutic options. We show here the poor prognostic value of high *PRMT5* expression in STS. Moreover, we demonstrate that the pharmacologic inhibition of PRMT5 has significant antitumor activity through the downregulation of glycolysis. Our findings support the clinical investigation of PRMT5 inhibition in STSs.

## Introduction

Soft-tissue sarcomas (STS) constitute a heterogeneous group of malignancies representing 1% of cancers in adults and 15% of cancers in children (1). Surgical resection is the cornerstone of treatment in the localized setting. However, despite adequate locoregional treatment, as many as 40% of patients develop metastatic disease. Doxorubicin is considered the standard first line of treatment for advanced STSs. However, its efficacy is modest, with an objective response rate of approximately 10% and a progression-free survival of less than 6 months (2). Therapeutic options are limited for patients with anthracycline-resistant disease. Overall, the prognosis of patients with advanced STS is extremely poor, with a median overall survival of less than 18 months (3). The identification of new therapeutic strategies is therefore an important clinical need.

Despite STS heterogeneity, a common genetic feature of STSs is their low mutation burden. Increasing evidence suggests that many STS cells exhibit epigenetic dysregulation, which plays a crucial role in tumorigenesis (4). Among several epigenetic mechanisms that can change chromatin compaction and thus lead to changes in gene expression in tumor cells, posttranslational methylation of histone arginine (R) residues plays an important role. Protein arginine methyltransferase 5 (PRMT5) methylates histone arginine residues [histone 3 arginine 8 (H3R8), histone 2A arginine 3 (H2AR3), and histone 4 arginine 3, (H4R3)], and these histone marks are associated with the transcriptional silencing of tumor suppressor genes such as RB1 and ST7 (5, 6) and differentiation-related genes in embryonic stem cells (7). PRMT5 plays a critical role in the cell cycle and proapoptotic effects of p53 via two mechanisms: direct inhibition of p53 activity via methylation of arginine residues in p53 and increased ubiquitination (and subsequent degradation) of p53 via differential splicing of murine double minute 4 (MDM4), a p53 ubiquitin ligase. Moreover, PRMT5 regulates several key cell signaling pathways through the methylation of EGFR and PI3K (8, 9). Increasing evidence suggests that PRMT5 is involved in tumorigenesis. PRMT5 protein is overexpressed in several epithelial tumors, including breast and lung cancer, as well as in hematologic malignancies and glial tumors (6, 10–12). PRMT5 overexpression alone is sufficient to transform normal fibroblasts (6, 10–12). Knocking down PRMT5 often leads to a decrease in cancer cell growth and survival. Specifically, increased PRMT5 expression and activity contribute to the silencing of several tumor suppressor genes in glioma cell lines (12).

To date, no data related to the biological role of PRMT5 inhibition or its potential as a treatment in STSs have been reported. The aim of our study was to investigate the antitumoral effects of PRMT5 inhibition in STSs and its related mechanisms of action.

## Materials and Methods

### Cell Culture and Reagents

The STS cell lines used in this study, called “IBxxx” or JR588, were derived from human STS surgically resected specimens after obtaining written, informed patient consent in accordance with the declaration of Helsinki and Bergonié Institute Institutional Review Board approval. Each in-house cell line was characterized by array comparative genomic hybridization performed on every 10 passage until p50 to verify that the genomic profile remained representative of the original tumor sample. No drift in the cell line maintenance or genetic imbalances were shown among the passages. IB112 and IB136 cells were derived from a leiomyosarcoma subtype tumor, IB111 and IB115 cells were from dedifferentiated liposarcoma cells, JR588 cells were undifferentiated pleomorphic sarcoma cells, IB114 cells were derived from a myxofibrosarcoma, and IB128 cells were derived from an extraskeletal osteosarcoma. The cells were grown in RPMI medium 1640 GlutaMAX Supplement (Life Technologies) in the presence of 10% (v/v) FBS and 1% penicillin/streptomycin (Thermo Fisher Scientific, Gibco) in flasks. The cells were maintained at 37°C in a humidified atmosphere containing 5% CO_2_. The cells were routinely passaged every 3 days, and all experiments were performed with cell lines between p25 and p60. Cell lines were periodically tested for *Mycoplasma* contamination by PCR (every 2 months).

GSK3326595, a potent, selective, reversible, and time-dependent inhibitor of PRMT5/methylosome protein 50 (MEP50; ref. 13), was provided by GlaxoSmithKline and prepared as a 20 mmol/L stock solution in DMSO and stored at −20°C for *in vitro* studies or prepared weekly in 100 mg/kg doses in 0.02 mol/L acetic acid for use *in vivo* studies.

### Survival Curves

For an analysis of metastasis-free survival according to the expression levels of PRMT5, expression and clinical data were obtained from the ATGsarc database (http://atg-sarc.sarcomabcb.org/; on-demand access), which integrates microarray data and clinical annotations from the French Sarcoma Group database. Survival rates were evaluated according to the Kaplan–Meier method using data obtained from the date of the initial diagnosis to the date of metastatic relapse or the latest follow-up. Significance values were calculated via log-rank tests, and *P* < 0.05 is considered to indicate a significant survival difference between groups.

### Cell Viability Assay

Depending on the doubling time, cells were seeded in triplicate at 200 to 1,000 cells/well in 96-well plates, cultured with fresh growth medium for at least 24 hours and treated with a range of increasing concentrations of GSK3326595 (GSK595) for 10 days (0.0001, 0.001, 0.01, 0.1, 1, 10, 25, and 50 µmol/L) with treatment renewal on day 3 and day 6. Cell viability was assessed by MTT [2-deoxyglucose (2-DG) and 3–4,5-dimethylthiazol-2-yl)-2,5-diphenyltetrazolium bromide] assay (Sigma-Aldrich) at a final concentration of 0.5 mg/mL and 3 hours of incubation. Then, the supernatant was discarded, 100 µL of DMSO was added, and the absorbance was read at 570 nm using a Flexstation 3 Plate reader (Molecular Devices) with 630 nm used as the reference wavelength. The IC_50_ was calculated with GraphPad Prism software version 5.0 for Windows (GraphPad Software, RRID: SCR_002798). Each experiment was repeated at least three times.

### Cell Proliferation and Clonogenic Assay

Depending on the doubling time, cells were seeded at 200 to 1,000 cells/well in four 96-well plates (proliferation assay) or 6-well plates (clonogenic assay), cultured with fresh growth medium for at least 24 hours and treated with or without GSK595 at the IC_50_ for 10 days with treatment renewal every 3 days. For the proliferation assay, after 3, 6, and 10 days in culture, cells were washed with PBS, trypsinized and resuspended in complete medium. An equal volume of resuspended cells from each well was transferred into a new 96-well plate and counted by FACS (FACSCalibur flow cytometer, BD Biosciences). A minimum of 6 wells per group that were untreated or treated with GSK595 were counted, and the experiment was repeated three times. For the clonogenic assay, after 10 days in culture, cells were washed with PBS, fixed for 10 minutes with 4% paraformaldehyde, and stained with crystal violet solution (HT90132, Sigma-Aldrich). After 15 minutes, the plates were rinsed with water and left to dry at room temperature.

### Western Blot Analysis

Cells were treated with or without GSK595 at IC_50_ every 3 days for 10 days. The cells were harvested in 100 µL of RIPA lysis buffer. The lysate was centrifuged (13,000 rpm, 15 minutes, 4°C), and the supernatant was stored at −20°C. Equal amounts of total protein (30 µg) were electrophoresed on 12% or 8% SDS polyacrylamide gels and transferred onto polyvinylidene difluoride membranes. The blots were probed overnight at 4°C with an anti-p53 (1:200 dilution, Santa Cruz Biotechnology, catalog no. sc-126, RRID:AB_628082), anti-actin (1:5,000 dilution, Sigma-Aldrich, catalog no. A3853, RRID:AB_262137), anti-p21 (1:1,000 dilution, Cell Signaling Technology, catalog no. 2947, RRID:AB_823586), anti-symmetric dimethyl arginine motif (SDMA; 1:1,000 dilution, Cell Signaling Technology, catalog no. 13222, RRID:AB_2714013), anti-GAPDH (1:2,000 dilution, Santa Cruz Biotechnology, catalog no. sc-51907, RRID:AB_629537), anti-PRMT5 (1:1,000 dilution, Cell Signaling Technology, catalog no. 79998, RRID:AB_2799945), anti-PCK2 (1:1,000 dilution, Cell Signaling Technology, catalog no. 8565, RRID:AB_11217628), anti-PFKM/PFKL (1:1,000 dilution, Abcam, catalog no. ab181064), anti-phospho-Rb (Ser807/811; 1:1,000 dilution, Cell Signaling Technology, catalog no. 8516, RRID:AB_11178658), anti-TIGAR (1:1,000 dilution, Cell Signaling Technology, catalog no. 14751, RRID:AB_2798596), anti-LDHD (1:1,000 dilution, Atlas Antibodies, catalog no. HPA041766, RRID:AB_2677660) primary antibodies diluted in PBST [DPBS 10X (Gibco) after 1X dilution; 0.1% Tween-20] with 5% BSA. A horseradish peroxidase–conjugated anti-mouse (GE Healthcare, catalog no. NA931, RRID:AB_772210) or anti-rabbit (GE Healthcare, catalog no. NA934, RRID:AB_772206) secondary antibody was diluted 1:5,000. The bound antibodies were visualized with a Fusion Fx7 imaging system (Fisher Bioblock Scientific) using an Immobilon Western enhanced chemiluminescence detection kit (Millipore Corporation). The resulting bands were analyzed and quantified using ImageJ 1.48v software (RRID:SCR_003070, NIH, Bethesda, MD).

### Animal Study

All animal experiments were performed with the approval of the Institutional Animal Use and Care Committee under project license APAFiS #20350-2019041812178984. This study followed the French and European Union guidelines for animal experimentation (RD 1201/05, RD 53/2013, and 86/609/CEE, respectively). IB115 or IB111 cells (3 × 10^6^ cells/200 µL) were inoculated subcutaneously into the right flank of 6 to 8 weeks old NSG mice procured and housed in institutional animal facilities. Once palpable, tumor volumes were calculated using the following formula: length × width^2^/2. Two (IB115 cells) or 3 weeks (IB111 cells) after implantation, when the average size of the tumors was approximately 100 mm^3^, the mice were randomly assigned to two groups (*n* = 9 mice per group) and treated orally with vehicle (0.02 mol/L acetic acid) or 100 mg of GSK595/kg body weight twice daily (5 days on/2 days off). Tumor size was measured every 2–3 days with calipers until a size of 2,000 mm^3^ was reached, after which the mice were euthanized. Tumor progression and weight, mouse body weight, and survival time were analyzed with GraphPad Prism software by two-way ANOVA and Bonferroni *post hoc* test.

### RNA Sequencing

For RNA sequencing (RNA-seq), IB111 cells were untreated or treated with GSK595 at the IC_50_ every 3 days for 10 days. RNA was then extracted using an RNeasy Plus Mini Kit (Qiagen) according to the supplier's instructions. The purity and concentration of the isolated RNA were determined with a NanoDrop spectrophotometer, and capillary electrophoresis using a Bioanalyzer 2100 (Agilent Technologies Inc.) was carried out to determine the RNA integrity number. RNA-seq was performed by IntegraGen, Inc. on a NovaSeq6000 S2 platform (Illumina Inc.) following the NEBNext Ultra II mRNA-seq library kit paired-end 100 × 2 read protocol. The number of paired-end reads produced by the sequencer was at least 30 M per sample. A bioinformatics analysis was performed as described previously (14). Briefly, the differential gene expression between groups of samples subjected to RNA-seq was performed using a statistical *t* test with the R package limma. We identified the significantly increased or decreased transcript levels with an FDR threshold of 0.05. Expression fold change was set to a minimum value of 2 to further filter the differentially expressed genes. For a gene set enrichment analysis (GSEA), the molecular signatures database (MSigDB) and fast gene set enrichment analysis (FGSEA) were used to identify hallmark pathways or Gene Ontology terms in which genes in an identified set were enriched.

### Metabolic Pathway Analysis

Four sarcoma cell lines (the IB111, IB114, IB115, and IB128 cells) were untreated or treated with GSK595 at IC_80_ every 3 days for 10 days. After RNA extraction and quality control (following the same protocol as used for RNA-seq), NanoString assays were performed using an nCounter Metabolic Pathways Panel in the nCounter Max Analyzer according to the manufacturer's instructions (NanoString Technology). Briefly, 300 ng of total purified RNA was hybridized with a Capture ProbeSet and Reporter CodeSets probes in a 15-µL reaction volume for 18 hours at 65°C. Hybridized samples were then immobilized on the nCounter cartridge in the nCounter Prep Station. The cartridge was ultimately scanned with 490 fields of view on the nCounter Digital Analyzer. Quality control assessment of the samples (imaging, binding density, positive control linearity, and limit of detection) was performed according to the NanoString recommendations. Raw gene expression was normalized using the RUVseq pipeline (15), and differential gene expression was analyzed using Deseq2 (v1.28). Pathway scoring was performed on normalized data using the single-sample GSEA method (ConsensusTME 0.0.1.9), and differences between vehicle and treated samples were evaluated for significance using Wilcoxon tests.

### Glucose Uptake and Lactate Production Assay

Glucose consumption and lactate production reflect the intracellular glycolysis of cells. To confirm the role of PRMT5 in glycolysis, we used a glucose uptake assay kit (Abcam, catalog no. ab136955) and a lactate colorimetric assay kit (Sigma-Aldrich, catalog no. MAK065) according to the protocols supplied by the manufacturers. Briefly, cells were seeded in a 96-well plate at 200 to 1,000 cells/well in 100 µL of culture medium, depending on the doubling time of each cell line. For glucose uptake, after 10 days of GSK595 treatment at the IC_80_ (renewed every 3 days), the cells were washed with PBS, and the growth medium was replaced with 100 µL of starvation medium (glucose- and FBS-free). After 1 hour of starvation, the medium was replaced with fresh starvation medium supplemented with 1 mmol/L 2-DG and incubated at 37°C for 20 minutes. The medium was carefully aspirated, and the cells were washed with PBS; then, 80 µL of extraction buffer was added to each well, and the plate was either stored at −80°C or an assay was immediately performed. The samples were diluted to 1/10 in the assay buffer before measurement. For lactate production, after 10 days of GSK595 treatment at the IC_80_ (renewed every 3 days), the supernatants were collected and diluted at 1/100. Lactate concentrations were determined by enzymatic colorimetric assay. For both glucose uptake and lactate production assays, cellular protein from each well was determined by DC Protein assay (Bio-Rad). The data were normalized against the total cell protein in each well.

### Data Availability

The data generated in this study have been deposited in NCBI's Gene Expression Omnibus (GEO) and are accessible through GEO Series accession numbers GSE243904 for RNA-seq experiment and GSE243759 for NanoString experiment. Any additional results can be obtained from the corresponding author.

## Results

### Prognostic Impact of PRMT5 Gene Expression

**FIGURE 1:**
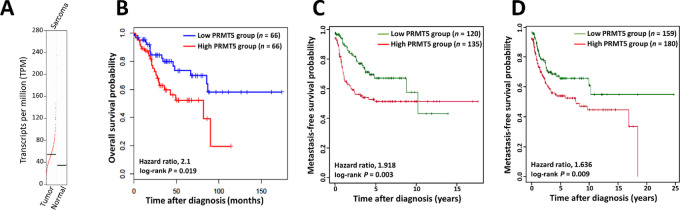
Prognostic impact of *PRMT5* gene expression in STSs. **A,** GEPIA dot plot showing *PRMT5* expression in sarcomas and normal samples, with each dot representing a distinct tumor (*n* = 262) or normal sample (*n* = 2). **B,** Kaplan‒Meier curves of overall survival according to *PRMT5* gene expression in 262 STSs from TCGA consortium (http://gepia.cancer-pku.cn/index.html). **C,** Kaplan‒Meier curves of metastasis-free survival in 255 patients with translocation-related sarcomas according to *PRMT5* expression (green line: low expression; red line: high expression: *n* = 135). **D,** Kaplan‒Meier curves of metastasis-free survival in 389 patients with complex genomic sarcomas according to *PRMT5* expression (green line: low expression, *n* = 159; red line: high expression: *n* = 180).

To evaluate the relative expression of the *PRMT5* gene in STS samples and normal tissue, we used a Gene Expression Profiling Interactive Analysis (GEPIA), a web-based interactive database that compiles the standardized analysis RNA-seq data from The Cancer Genome Atlas (TCGA) and GTEx databases (16). We found that *PRMT5* was significantly upregulated in tumor tissues and that high *PRMT5* gene expression was significantly associated with worsened overall survival (log-rank test, *P* = 0.019; Fig. 1A and B). To confirm the prognostic value of *PRMT5* expression, we analyzed the transcriptome data from 255 translocation-related STS samples and 389 complex genomic STS samples with annotated clinical data from the French Sarcoma Group (Fig. 1C and D). High *PRMT5* gene expression levels were significantly associated with worsened metastasis-free survival in both the translocation-related and complex genomics STS datasets (log-rank test, *P* = 0.003 and *P* = 0.009, respectively), supporting the relevance of PRMT5 as a potential therapeutic target in this indication.

### Antitumor Activity of PRMT5 Inhibition in STSs

**FIGURE 2:**
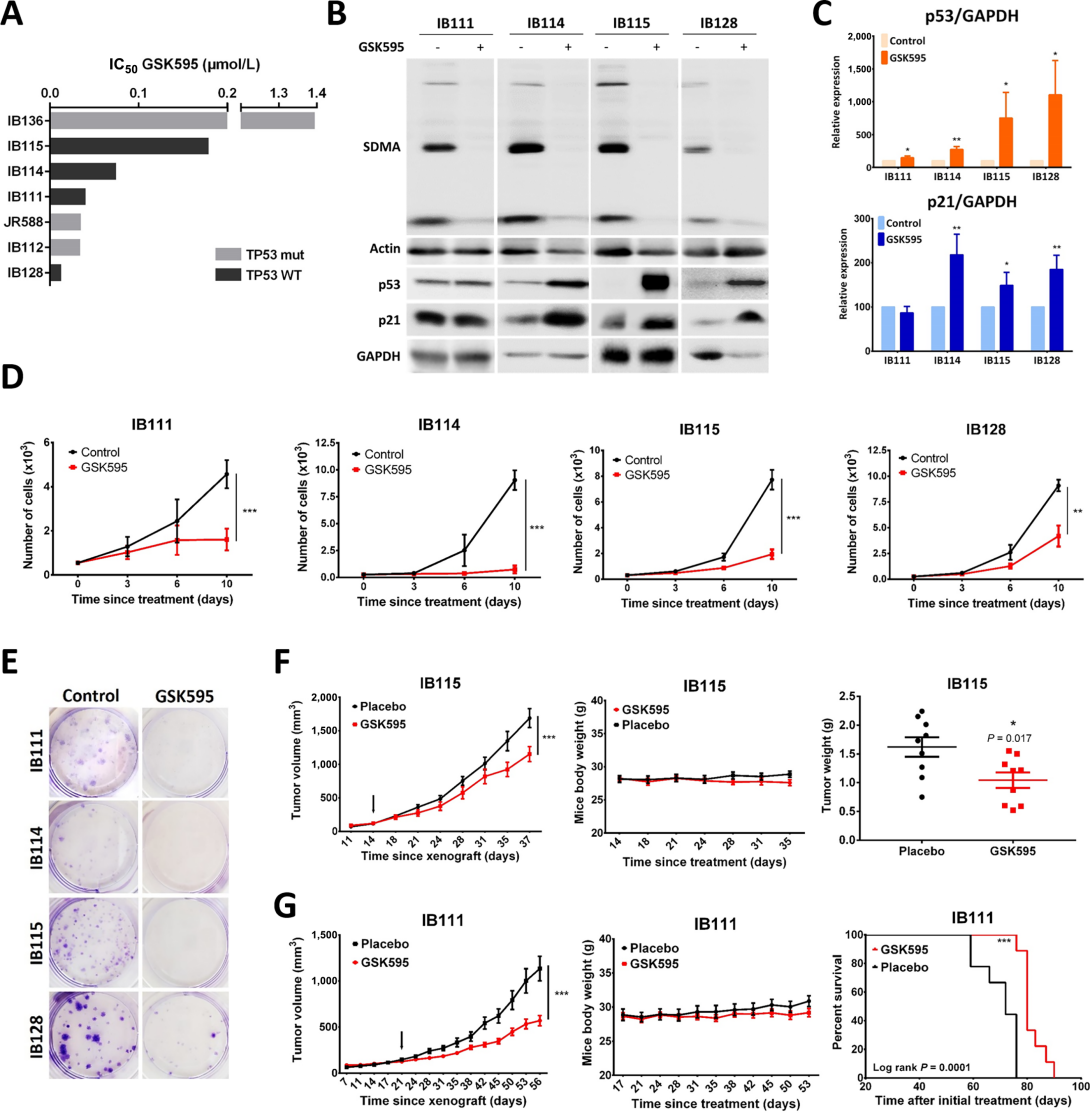
Antitumor effects of PRMT5 inhibition on STSs. **A,** Assessment of cell viability with PRMT5 inhibitor GSK3226595 (GSK595) in seven STS cell lines (with TP53 deleted or not) as determined by MTT assay (*n* = 3 or more). Cells were treated with a range of increasing concentrations of drug for 10 days and the IC_50_ was calculated with GraphPad Prism software. **B,** Western blot analysis showing of SDMA, p53, and p21 protein expression in four STS cell lines after 10 days of GSK595 treatment at the respective IC_50_. **C,** Quantitation of p53 and p21 bands in B using ImageJ software. GAPDH served as the loading control (*n* = 3). **D,** Proliferation assay after PRMT5 inhibition: Cells were untreated or treated with GSK595 at the IC_50_ and counted after 3, 6, and 10 days by flow cytometry. Fresh medium with drug treatment at the appropriate concentration was used to replace the medium every 3 days (*n* = 3; **, *P* < 0.01; ***, *P* < 0.001 two-way ANOVA and Bonferroni multiple comparisons test). **E,** Clonogenic assay: Representative images of stained cells (crystal violet) after 10 days of GSK595 treatment at the IC_80_ (*n* = 2). **F,** Efficacy of the PRMT5 inhibitor *in vivo* in IB115 xenograft. IB115 cells were used to establish xenograft tumors in NSG mice (*n* = 9 mice per group), and the tumors were treated with vehicle or GSK595 at 100 mg/kg orally twice daily (5 days-on/2 days-off; ***, *P* < 0.001 two-way ANOVA and Bonferroni multiple comparisons tests, arrow = start of treatment). Tumor growth, body weight, and final tumor weight were measured. **G,** Efficacy of the PRMT5 inhibitor *in vivo* in IB111 xenograft mice. IB111 cells were used to establish xenograft tumors in NSG mice, which were treated with the same concentrations as the IB115 cells. Tumor growth and body weight were measured, and the tumors were monitored until the end of the treatment period or until they reached 2,000 mm^3^ (day of sacrifice); the data were used to draw the survival curve.

To investigate the antitumor effect of the pharmacologic inhibition of PRMT5 on STS, a panel of seven cell lines representative of several histologic subtypes were treated with increasing concentrations of GSK595 for 10 days. The results showed that GSK595 suppressed the viability of all seven STS cell lines with IC_50_ values ranging from 0.01 to 1.4 µmol/L (Fig. 2A). Because of its methyltransferase function, PRMT5 generates the majority of cellular SDMA; therefore, we examined global symmetric dimethylarginine level as a readout of PRMT5 enzyme activity. As shown in Fig. 2B, GSK595 treatment decreased the SDMA level, indicating the on-target effects of the drug without any changes to the basal level of PRMT5 (Supplementary Fig. S1A). This decrease in global SDMA level aligned with a significant increase in p53 and p21 protein levels (Fig. 2B and C, except in IB111 cells for the p21 level measurement). Notably, despite the increase in p53 level, the sensitivity of STS cells to GSK595 can be independent of TP53 expression since even TP53-mutated or -deleted cells, such as IB112 and JR588 cells, were characterized by very low IC_50_ values (Fig. 2A). To understand the mechanisms of cell viability diminution, we measured the apoptosis rate and found that GSK595 exerted a negligible or weak cytotoxic effect, with less than 20% of apoptotic cells detected and only in two cell lines (Supplementary Fig. S1B). Given the role of TP53 in apoptosis, this lack of cell death may be tied to the lack of sensitivity to TP53 expression. However, a profound decrease in proliferation was observed for all STS cell lines *in vitro*, and a decrease in colony formation capacity after GSK595 treatment was also observed (Fig. 2D and E). Surprisingly, no effect on cell-cycle distribution or cyclin and cyclin-dependent kinase expression was observed after GSK595 treatment (Supplementary Fig. S1C and S1D). To investigate the *in vivo* antitumor effect, two NSG xenograft mouse models of liposarcoma (IB111 and IB115 cells) were established. Once the tumors were palpable, the mice were treated with GSK595 (or vehicle) at 100 mg/kg orally twice daily. As shown in Fig. 2F and G, tumor growth was significantly decreased in both mouse models, and the mouse survival was longer in the GSK595-treated group. No sign of toxicity (e.g., body weight loss) in the mice was found, confirming the therapeutic potential of PRMT5 inhibition in the STS context.

### Transcriptome Analysis of PRMT5 Inhibition in STSs

**FIGURE 3:**
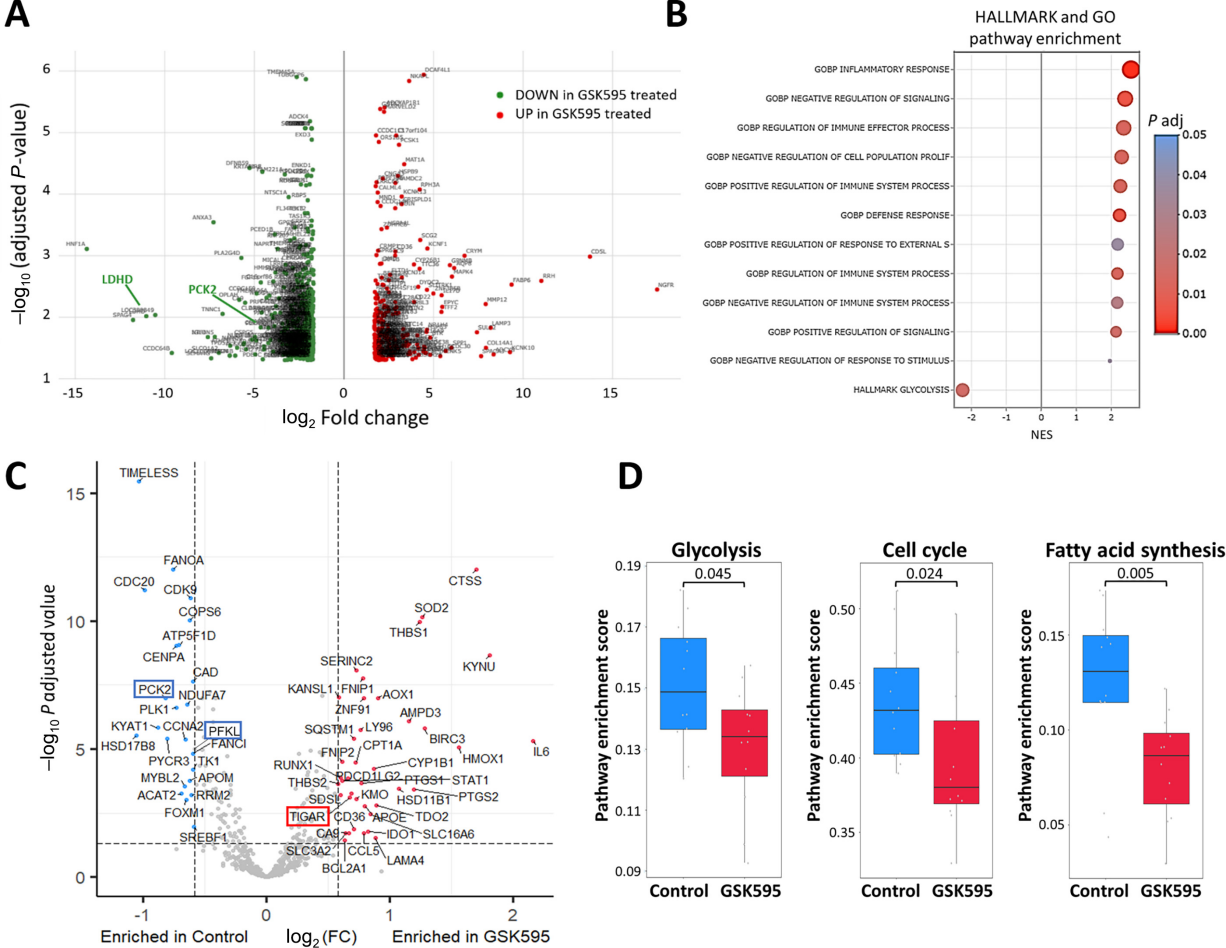
Transcriptome analysis of PRMT5 inhibition in STSs. **A,** Volcano plot showing differential gene expression after RNA-seq with the IB111 cell line that was untreated or treated with GSK595 for 10 days at the IC_50_ in biological triplicates. A total of 556 genes (green dots) and 486 genes (red dots) were significantly downregulated or upregulated after treatment. **B,** Pathway analysis of RNA-seq shown in A. MSigDB and FGSEA were used to identify hallmark pathways or Gene Ontology (GO) biological process terms in which the genes of an identified set were enriched. **C** and **D**, Four sarcoma cell lines (the IB111, IB114, IB115, and IB128 cells lines) were untreated or treated with GSK595 for 10 days at IC_80_ in triplicate, and their transcriptomes were analyzed using an nCounter Metabolic Pathways Panel from NanoString. **C,** Volcano plot showing differentially expressed genes in the panel. Blue dots and red dots represent significantly downregulated and upregulated genes after treatment, respectively. Gray dots represent nonsignificantly regulated genes. **D,** The three pathways showing the highest gene enrichment as determine with NanoString gene signatures following the single-sample GSEA method.

To decipher the mechanism of action of GSK595 in the STS cell lines, we explored the transcriptomic changes in IB111 cells after 10 days of treatment by RNA-seq. We found that 1,042 genes were differentially expressed after PRMT5 inhibition (Fig. 3A). Hallmarks and Gene Ontology gene sets obtained from the MSigDB were used to identify enriched pathways (Fig. 3B). This analysis clearly showed transcriptional downregulation of the glycolysis pathway in IB111 cells after treatment with GSK595. To characterize the impact of PRMT5 inhibition on glucose metabolism in STS cells in greater depth, we analyzed the gene expression profile of three additional cell lines in addition to the IB111 cell lines (the IB114, IB115, and IB128 cell lines) by using an nCounter Metabolic Pathways Panel assay. The combined analysis of the four cell lines led to the identification of 82 genes differentially expressed after treatment with GSK595 (32 and 50 genes upregulated and downregulated, respectively; Fig. 3C; the complete gene list is provided in a Supplementary Excel file). Cell-cycle progression and fatty acid synthesis pathway activity, as well as glycolysis pathway activation, were downregulated (Fig. 3D), confirming the RNA-seq results obtained with IB111 cells.

### PRMT5 Inhibition Impairs Glycolysis in STSs

**FIGURE 4:**
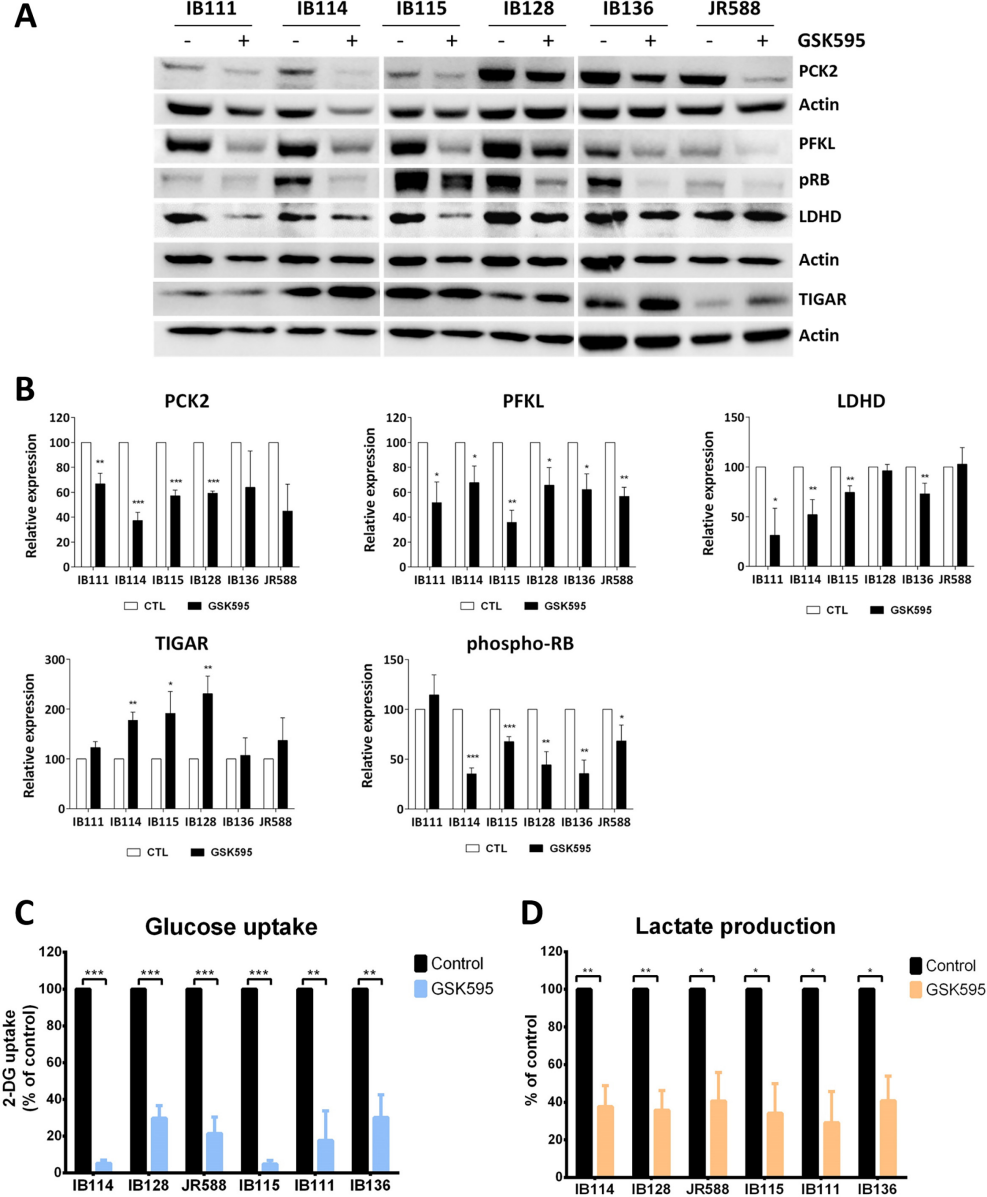
PRMT5 inhibition impairs glycolysis in STSs. **A,** Western blot analysis showing key proteins involved in glycolysis. Sarcoma cell lines were treated for 10 days without or with GSK595 at the IC_80_ before protein extraction. **B,** Quantitation of Western blot bands (*n* = 2 or more). **C** and **D,** Relative glucose uptake and lactate production were measured by colorimetric assay after 10 days of treatment with GSK595 at the IC_80_. All values were normalized to the total amount of protein (*n* = 3 or more; *, *P* < 0.05; **, *P* < 0.01; ***, *P* < 0.001, multiple *t* test using the Holm‒Sidak method).

**FIGURE 5:**
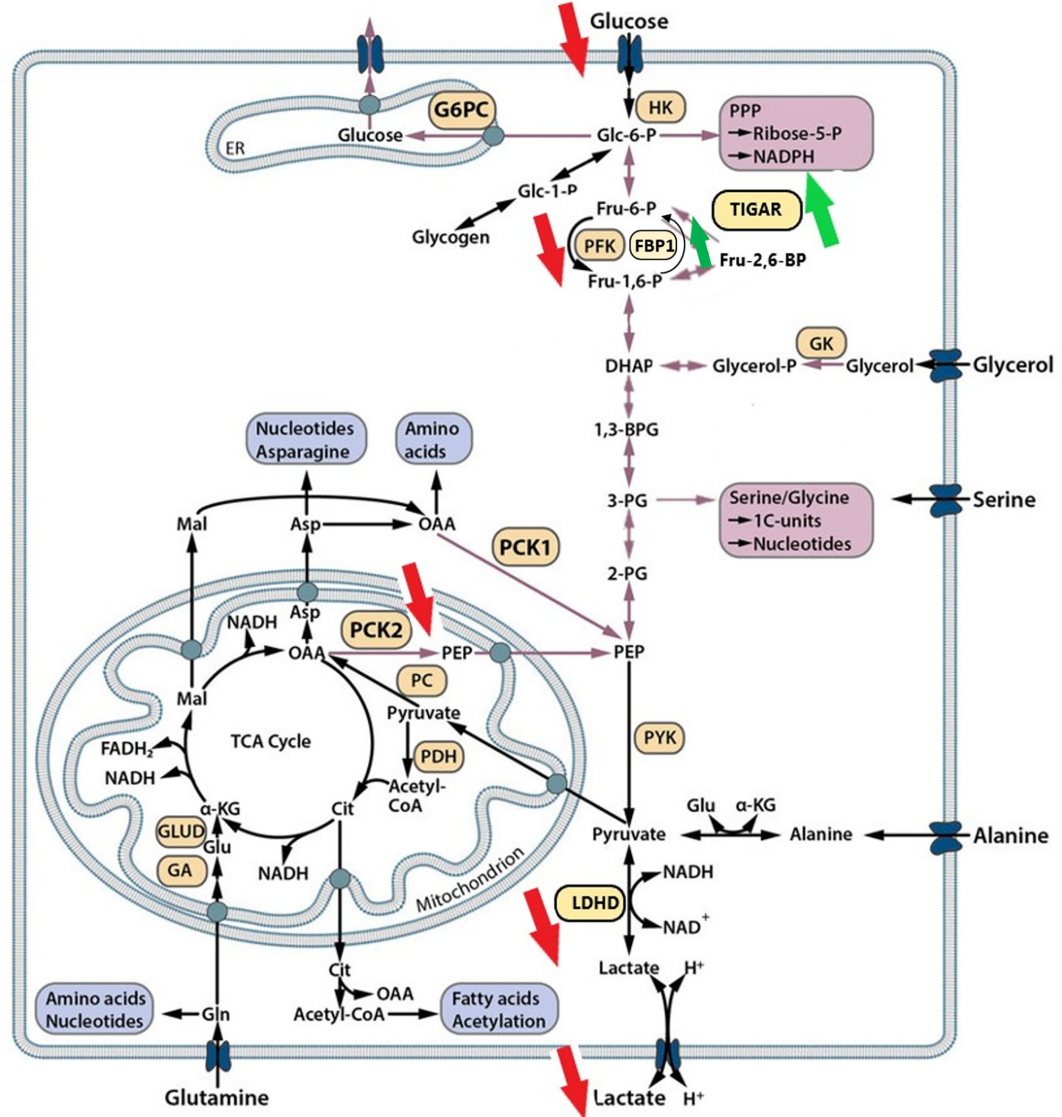
Schematic representation of glycolysis impaired by PRMT5 inhibition in STSs. The red and green arrows show the decrease and increase observed upon GSK595 treatment, respectively. Adapted from Grasmann et al. Biochim Biophys Acta Rev Cancer. 2019. G6PC, glucose-6-phosphatase; HK, hexokinase; PFK, phosphofructokinase; FBP1, fructose-1,6-bisphosphatase 1; TIGAR, TP53-inducible glycolysis and apoptosis regulator; PPP, pentose phosphate pathway; GK, glycerol kinase; PCK1, cytosolic isoform of phosphoenolpyruvate carboxykinase; PCK2, mitochondrial isoform of phosphoenolpyruvate carboxykinase; PC, pyruvate carboxylase; PDH, pyruvate dehydrogenase; PYK, pyruvate kinase; GLUD, glutamate dehydrogenase; GA, glutaminase; LDHD, D-lactate dehydrogenase; and TCA cycle, tricarboxylic acid cycle.

Then, we validated the aforementioned results by evaluating four regulated genes encoding key enzymes involved in glucose metabolism via Western blotting: phosphofructokinase, liver type (PFKL), D-lactate dehydrogenase (LDHD), phosphoenolpyruvate carboxykinase 2 (PCK2), and TP53-inducible glycolysis and apoptosis regulator (TIGAR). All the cell lines showed decreased protein expression of PFKL, LDHD, and PCK2 and an increase in the TIGAR protein level after PRMT5 inhibition with GSK595. These regulatory effects were found to be concomitant with a decrease in the phospho-Rb level, which is used as a marker of cell proliferation (Fig. 4A and B). To validate the impact of PRMT5 inhibition on aerobic glycolysis, we measured glucose uptake and lactate production in the culture medium of the STS cells exposed to GSK595. As shown in Fig. 4C and D, all the cell lines tested exhibited a profound decrease in both glucose uptake and lactate level after GSK595 treatment. Taken together, these results demonstrate that PRMT5 inhibition by GSK595 impairs glycolysis in STSs at several levels, as schematically represented in Fig. 5.

## Discussion

PRMT5 overexpression has been associated with poor overall survival in several nonmesenchymal tumors (11, 17). By analyzing two large independent datasets, we show that PRMT5 overexpression is also associated with an increased risk of metastasis and adverse outcomes in patients with STS, suggesting important roles in sarcoma tumorigenesis and a potential role as a therapeutic target.

We confirmed the therapeutic potential of PRMT5 by showing that pharmacologic inhibition led to a strong antiproliferative effect both *in vitro* and *in vivo*. Interestingly, we found that a signature acquisition connected to glycolysis was significantly blocked by GSK595, with the expression of genes encoding PFKL, LDHD, and PCK2 notably inhibited. Cell metabolism reprogramming is a crucial hallmark of cancer. PFKL, an isoform of PFK, is the first committed step of glycolysis, in which fructose-6-phosphate (F6P) is irreversibly converted to fructose-1,6-bisphosphate (F1,6BP; ref. 18). Notably, FBP1, which exerts an effect opposite that of PFK in regulating F1,6BP, as well as TIGAR, was found to be upregulated after GSK595 treatment, as shown in the transcriptome analysis of the IB111 cell line (Fig. 5). Downstream of this pathway, LDHD, a lactate dehydrogenase isoform, catalyzed the reduction of pyruvate to lactate, the final product in the glycolysis pathway (19). Finally, PCK2 (or PEPCK-M) encodes the mitochondrial form of PCK and catalyzes the conversion of OAA (oxaloacetate) to PEP (phosphoenolpyruvate), which is a rate-limiting step in gluconeogenesis. PCK2 has recently been shown to mediate a metabolic shunt in response to glucose deprivation, supplying carbon derived from glutamine, not glucose, to generate the glycolytic pathway intermediates required for biosynthesis and cell proliferation (20). Thus, the regulation of glycolytic enzymes essential for energy production seems to be the major mechanism underlying the GSK595-mediated suppression of STS progression.

Qin and colleagues (21) showed that PRMT5 promoted glycolysis in pancreatic cancer through the F-box/WD repeat-containing protein 7 (FBW7)/cMyc axis. Han and colleagues (22) demonstrated that PRMT5 enhanced aerobic glycolysis by regulating the liver X receptor α (LXRα)/NFκBp65 pathway in breast tumor cells. In this study, we revealed a crucial role for PRMT5 in promoting STS cell proliferation by regulating glycolytic flux, suggesting that the effect of PRMT5 on metabolism is not dependent of a specific tumor type.

Several PRMT5 inhibitors are being developed in clinical studies (23–26). The largest clinical trial reported thus far is the METEOR-1 study, which is a phase I study designed to investigate the safety and efficacy of the PRMT5 inhibitor GSK3326595 in advanced solid tumors. A total of 218 patients were enrolled in the study (23). The safety profile was found to be manageable, with the most frequent adverse events being fatigue, anemia, nausea, and thrombocytopenia. The observed results appear consistent with other published data for PRMT5 inhibitors (24–26). Preliminary signs of activity were observed in patients with adenoid cystic carcinoma, non–Hodgkin lymphoma, head and neck cancer, non–small cell lung cancer, and ovarian cancer. Among all 355 patients enrolled in the phase I studies investigating PRMT5 inhibitors, only 5 presented with a sarcoma, and 2 of these patients achieved long-term disease stabilization (>6 months).

One limitation of our study is that *in vivo* experiments were conducted in a restricted number of models. Because of the heterogeneity of STS, our findings may or may not be applicable to different sarcoma subtypes. Nevertheless, our results suggest that PRMT5 inhibition warrants further investigation in patients with STS. Additional studies in diverse sarcoma subtypes will be essential to confirm and expand upon our findings.

## Supplementary Material

Supplementary Methods supplementary methods for apoptosis and cell cycle analysis and Western blot

Supplementary Figure 1 and legend Suppl Fig 1 showing the PRMT5 inhibition effect on PRMT5 expression, cell cycle phases and apoptosis
