# Supplementary material for: Antitumor Effects of PRMT5 Inhibition in Sarcomas
Source: Cancer Res Commun. 2023 Nov 2;3(11):2211–20. doi: 10.1158/2767-9764.CRC-23-0239 (PMC10621483; doi:10.1158/2767-9764.CRC-23-0239)
Supplement: Supplementary Methods — for apoptosis and cell cycle analysis and Western blot [file crc-23-0239-s01.docx]

**Supplementary Methods for:**

# Anti-tumor activity of PRMT5 inhibition in sarcomas

Verbeke Stéphanie^1,2^, Bourdon Aurélien^1,2^, Guegan Jean-Philippe^3^, Leroy Laura^1,2^, Chaire Vanessa^1,2^, Richard Elodie^4^, Bessede Alban^3^, Italiano Antoine^1,2,5^.

**Affiliations:**

1. Sarcoma Unit, Bergonié Institute, 33000 Bordeaux, France
2. INSERM U1312 BRIC BoRdeaux Institute of onCology, University of Bordeaux, 33000 Bordeaux, France
3. Explicyte, 33000 Bordeaux, France
4. Service Commun des Animaleries, University of Bordeaux, 33000 Bordeaux, France
5. Faculty of Medicine, University of Bordeaux, 33000 Bordeaux, France

**Corresponding author**

Pr Antoine ITALIANO

Institut Bergonié, 229 cours de l’Argonne, 33000 Bordeaux, France

Email: [a.italiano@bordeaux.unicancer.fr](mailto:a.italiano@bordeaux.unicancer.fr)

Phone: + 33 5 47 30 60 88

# Supplementary Methods

**Cell cycle and apoptosis assay**

STS cells (5 × 10^4^ cells/well) were seeded in 6-well plates in triplicate. After 24 h, cells were treated with GSK595 at the IC_50_ on day 0, day 3 and day 6. After 6 days of treatment, cell cycle and apoptosis were analyzed by flow cytometry (FACS Calibur flow cytometer, BD Biosciences, San Jose, CA, USA). For apoptosis assessment, cells were washed once with phosphate-buffered saline (PBS) and labeled with annexin-V-FITC and propidium iodide (PI) according to the manufacturer’s protocol (BD Biosciences, San Jose, CA, USA). Then, the percentage of cells in early apoptosis (annexin-V-positive, PI-negative) and in late apoptosis or necrosis (annexin-V and PI-positive) was calculated using FlowJo version 7.6.3 (RRID: SCR_008520). The percentages of overall death (sum of early and late apoptosis) are represented as the mean ± SEM values based on 2 independent experiments.

For cell cycle analysis, cells were permeabilized with 70% ethanol at -20 °C overnight. Following ethanol removal, the cells were washed with PBS and stained with a PI and ribonuclease-containing solution before measuring DNA content by FACS. The data were analyzed with FlowJo software, and the results were expressed in terms of the percentage of cells in a given phase of the cell cycle based on 2 or 3 independent experiments depending on the cell lines.

**Western blot**

Cells were treated with or without GSK595 at IC_50_ every 3 days for 10 days. The cells were harvested in 100 μL of radio-immuno-precipitation assay (RIPA) lysis buffer. The lysate was centrifuged (13 000 rpm, 15 min, 4°C), and the supernatant was stored at -20°C. Equal amounts of total protein (30 μg) were electrophoresed on 12% or 8% sodium dodecyl sulfate polyacrylamide gels and transferred onto polyvinylidene difluoride membranes. The blots were probed overnight at 4°C with an anti-actin (1:5000 dilution, Sigma-Aldrich Cat# A3853, RRID:AB_262137), anti- Symmetric Di-Methyl Arginine Motif (SDMA) (1:1000 dilution, Cell Signaling Technology Cat# 13222, RRID:AB_2714013), anti-PRMT5 (1:1000 dilution, Cell Signaling Technology Cat# 79998, RRID:AB_2799945), anti-Cyclin D1 (1:1000 dilution, Cell Signaling Technology Cat# 2978, RRID:AB_2259616), anti-Cyclin D3 (1:1000 dilution, Cell Signaling Technology Cat# 2936, RRID:AB_2070801), anti-Cyclin B1 (1:1000 dilution, Thermo Fisher Scientific Cat# MA1-155, RRID:AB_2536863), CDK4 (1:1000 dilution, Cell Signaling Technology Cat# 12790, RRID:AB_2631166), CDK2 (1:1000 dilution, Cell Signaling Technology Cat# 2546, RRID:AB_2276129) primary antibody diluted in PBST (DPBS 10X (GibcoTM) after 1X dilution; 0.1% Tween-20) with 5% bovine serum albumin. The horseradish peroxidase-conjugated secondary antibody anti-mouse (GE Healthcare Cat# NA931, RRID:AB_772210) or anti-rabbit (GE Healthcare Cat# NA934, RRID:AB_772206) was diluted at 1:5000. Bound antibodies were visualized on Fusion Fx7 imaging system (Fisher Bioblock Scientific, Waltham, USA) using the ImmobilonTM Western enhanced chemiluminescence detection kit (Millipore Corporation, Billerica, USA).
