# Supplementary material for: Antitumor Effects of PRMT5 Inhibition in Sarcomas
Source: Cancer Res Commun. 2023 Nov 2;3(11):2211–20. doi: 10.1158/2767-9764.CRC-23-0239 (PMC10621483; doi:10.1158/2767-9764.CRC-23-0239)
Supplement: Supplementary Figure 1 and legend — Suppl Fig 1 showing the PRMT5 inhibition effect on PRMT5 expression, cell cycle phases and apoptosis [file crc-23-0239-s02.docx]

**Supplementary Figure and Legend for:**

# Anti-tumor activity of PRMT5 inhibition in sarcomas

Verbeke Stéphanie^1,2^, Bourdon Aurélien^1,2^, Guegan Jean-Philippe^3^, Leroy Laura^1,2^, Chaire Vanessa^1,2^, Richard Elodie^4^, Bessede Alban^3^, Italiano Antoine^1,2,5^.

**Affiliations:**

1. Sarcoma Unit, Bergonié Institute, 33000 Bordeaux, France
2. INSERM U1312 BRIC BoRdeaux Institute of onCology, University of Bordeaux, 33000 Bordeaux, France
3. Explicyte, 33000 Bordeaux, France
4. Service Commun des Animaleries, University of Bordeaux, 33000 Bordeaux, France
5. Faculty of Medicine, University of Bordeaux, 33000 Bordeaux, France

**Corresponding author**

Pr Antoine ITALIANO

Institut Bergonié, 229 cours de l’Argonne, 33000 Bordeaux, France

Email: [a.italiano@bordeaux.unicancer.fr](mailto:a.italiano@bordeaux.unicancer.fr)

Phone: + 33 5 47 30 60 88


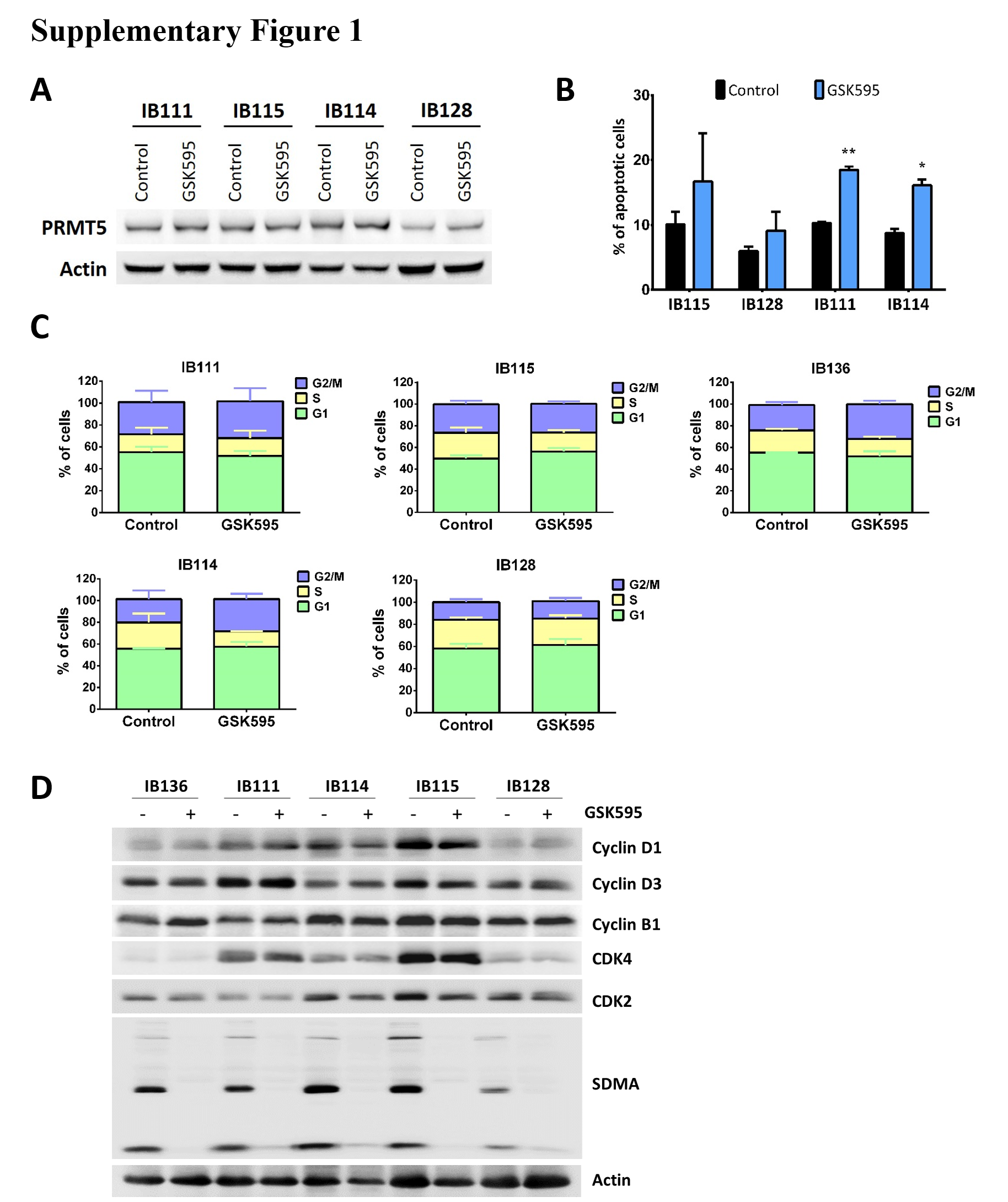


**Supplementary Figure 1.**

**PRMT5 inhibition effect on PRMT5 expression, cell cycle phases and apoptosis.** STS cell lines were treated or not with GSK595 at their respective IC50 for 6 days. **A,** Western blot of PRMT5, with Actin used as the loading control. **B,** Apoptosis was assessed by flow cytometry after Annexin-V-FITC and propidium iodide staining. Percentage of apoptotic cells is the sum of Annexin-V positive cells (early apoptosis) and the double stained Annexin-V and PI positive cells (late apoptosis). (n = 2, *P < 0.05, **P < 0.01 multiple t-test using the Holm-Sidak method). **C,** Cell cycle analysis was measured by flow cytometry after propidium iodide staining (IB115 and 128: n = 3, IB136, 114, 111: n = 2). **D,** Western blot of cyclins and CDKs were performed after 10 days of STS cell lines treatment with or without GSK595 at their IC50. Actin was used as loading control and SDMA as on-target effect control (n = 2).
